# Prevalence of clinically actionable disease variants in exceptionally long-lived families

**DOI:** 10.1186/s12920-020-0710-5

**Published:** 2020-04-10

**Authors:** Paige Carlson, Mary K. Wojczynski, Todd Druley, Joseph H. Lee, Joseph M. Zmuda, Bharat Thyagarajan

**Affiliations:** 1 University of Minnesota Medical School, Duluth, MN USA; 2 Division of Statistical Genomics, Department of Genetics, Washington University School of Medicine, St. Louis, MO USA; 3 Center for Genome Sciences and Systems Biology, Washington University School of Medicine, 660 South Euclid Avenue, Campus Box 8116, St. Louis, MO 63108 USA; 4 Department of Pediatrics, Washington University School of Medicine, 660 South Euclid Avenue, Campus Box 8116, St. Louis, MO 63108 USA; 5 Sergievsky Center, College of Physicians and Surgeons, Columbia University New York, New York, NY USA; 6 Taub Institute, College of Physicians and Surgeons, Columbia University New York, New York, NY USA; 7 Departments of Epidemiology and Neurology, Columbia University New York, New York, NY USA; 8 Department of Epidemiology, University of Pittsburgh Graduate School of Public Health, Pittsburgh, PA USA; 9 Department of Laboratory Medicine and Pathology, University of Minnesota, MMC 609, 420 Delaware street, Minneapolis, MN 55455 USA

**Keywords:** Incidental genetic findings, Long lived families, Population studies, Pathogenic variants

## Abstract

**Background:**

Phenotypic expression of pathogenic variants in individuals with no family history of inherited disorders remains unclear.

**Methods:**

We evaluated the prevalence of pathogenic variants in 25 genes associated with Mendelian-inherited disorders in 3015 participants from 485 families in the Long Life Family Study (LLFS). Boot-strapping and Fisher’s exact test were used to determine whether allele frequencies in LLFS were significantly different from the allele frequencies reported in publicly available genomic databases.

**Results:**

The proportions of pathogenic autosomal dominant mutation carriers in *BRCA1* and *SDHC* in LLFS study participants were similar to those reported in publicly available genomic databases (0.03% vs. 0.0008%, *p* = 1 for *BRCA1*, and 0.08% vs. 0.003%, *p* = 0.05 for *SDHC*). The frequency of carriers of pathogenic autosomal recessive variants in *CPT2*, *ACADM*, *SUMF1*, *WRN*, *ATM*, and *ACADVL* were also similar in LLFS as compared to those reported in genomic databases. The lack of clinical disease among LLFS participants with well-established pathogenic variants in *BRCA1* and *SDHC* suggests that penetrance of pathogenic variants may be different in long lived families.

**Conclusion:**

Further research is needed to better understand the penetrance of pathogenic variants before expanding large scale genomic testing to asymptomatic individuals.

## Introduction

Next generation sequencing (NGS) has revolutionized the genetic diagnosis for several diseases such as hearing loss, vision loss, cardiovascular disorders, and neurodegenerative disorders through testing of single genes, targeted gene panels, and whole exome sequencing [1, 2]. Large scale sequencing efforts are increasingly being used in several research studies that include participants in the general population without a strong family history or with subtle clinical presentations [3]. Large scale sequencing effort such as the 1000 Genomes Project [4] and 6500 Exome Sequencing Project (ESP) [5] have demonstrated that phenotypically healthy individuals harbor variants previously classified as pathogenic, calling into question the pathogenicity of these variants. This misclassification may be due to initial classification of these variants being based on studies with limited sample size or incomplete penetrance of specific pathogenic variants [6]. The American College of Medical Genetics and Genomics (ACMG) has published a specific list of medically actionable genes known to cause autosomal dominant conditions in order to provide guidance on return of secondary genetic findings to patients and/or their care-givers when undergoing whole genome and whole exome sequencing in the context of clinical sequencing [7, 8]. However, the variable penetrance of rare variants, particularly in the absence of relevant family history, remains a major limitation in expanding large scale sequencing efforts beyond the narrowly defined clinical settings into the general population.

Studies on centenarians are particularly informative in this regard as variants previously classified as pathogenic but found in these individuals are less likely to be a pathogenic variant that causes highly penetrant monogenic Mendelian diseases that typically have an early onset of disease [9, 10]. A previous study on 44 Ashkenazi Jewish centenarians who underwent whole genome sequencing identified over 130 pathogenic or likely pathogenic variants present in the centenarians’ genomes; the authors concluded that mutations previously classified as pathogenic might not necessarily preclude a long life [11].

Since this study was limited to 44 centenarians of Ashkenazi Jewish descent, we evaluated the prevalence of established pathogenic and likely pathogenic variants in long-lived families of broad European descent in Long Life Family Study (LLFS), a family-based cohort study designed to evaluate genetic and environmental factors associated with exceptional survival to extend the generalizability of the previous study findings to a broader population. In addition, the family-based design of LLFS also allowed us to evaluate segregation patterns of pathogenic mutations within families. This study analyzed sequences from 25 loci associated with hereditary disorders and compared the prevalence of pathogenic variants in LLFS participants vs. a general population of predominantly European ancestry found in publicly available genomic databases of germline variants.

## Methods

### Study population

LLFS enrolled long-lived probands and their siblings (*n* = 1445; baseline age: 91 ± 8 years), their offspring (*n* = 2346; baseline age: 61 ± 8 years) and spousal controls (*n* = 785; baseline age 62 ± 8 years) from three U.S. field centers (New York City, Boston, and Pittsburgh) and one Danish field center. The study design and selection criteria have been described previously [12, 13]. Included in this analysis were 3015 participants from 485 families (including spousal controls) in three US field centers. Danish participants were excluded as Danish participants did not have appropriate consent for participation in this genetic study. Participants of non-European ancestry were also excluded to minimize issues related to variant classification in non-Caucasian populations.

### Sequencing of variants

The LLFS participants had 464 genes sequenced that were selected collectively by the LLFS investigators due to their published association with age-related phenotypes [14]. Of these 464 genes, 25 genes associated with Mendelian forms of metabolic defects, familial cardiovascular disorders, familial cancer predispositions, and familial neurodegenerative disorders (Supplementary Table 1) were included for further analysis in this study. This included seven genes on the ACMG list of secondary genetic findings and the remaining 18 genes associated with autosomal recessive conditions were included to evaluate whether rates carrier status for Mendelian diseases were different in LLFS families as compared to the general population.

### Variant classification

We used ANNOVAR [15] to annotate the genetic variants. The prevalence of the annotated variants was found using public genome variant databases such as gnomAD v2 [16], which consists of exome and genome data from 141,456 individuals sequenced as part of various disease-specific and population genetic studies. Approximately 55% of individuals included in gnomAD v2 were of European ancestry and 46% of individuals were women. All variants with an allele frequency < 0.5% in the general population were further evaluated manually and classified into five categories using the ACMG criteria: pathogenic, likely pathogenic, uncertain significance, likely benign, and benign [17, 18]. In addition to public genome variant databases such as those listed above, we used publicly available databases of clinically relevant genetic variants such as ClinVar [19] to determine if the rare variants had been previously classified as pathogenic or likely pathogenic by other clinical laboratories. Variants not previously classified in ClinVar were evaluated using locus/disease specific databases (LOVDs). If the variant was not classified in ClinVar or LOVDs, we conducted a literature search in PubMed using common nomenclatures, such as dbSNP reference numbers, gene specific mutations, and chromosome position, to identify peer-reviewed publications that had identified the mutations.

In silico predictions were obtained using MutationTaster [20], GERP [21], phyloP100way [22], LRT [23], SIFT [24], and PolyPhen-2 [25]. If three or more of these programs listed the variant as pathogenic or conserved, then the criterion for PP1 was met. We used GERP scores of greater than 3.0 to be pathogenic [5]. We utilized an online genetic interpretation tool from the University of Maryland School of Medicine to assist with and standardize variant classification using the ACMG criteria [26]. All variants classified as pathogenic/likely pathogenic were manually reviewed and confirmed by a molecular and genetic pathologist (BT).

### Statistical analysis

We used Fisher’s exact test to compare the allele frequencies in LLFS and the general population of European descent in the gnomAD database [16]. Since LLFS is a family-based study, the observed allele frequencies were skewed by the prevalence of genetic variants within individual families. To obtain an estimate of the population frequency of variants in LLFS, we estimated the allele frequency in LLFS by randomly selecting one family member from the LLFS families, repeating the procedure 1000 times to determine an average estimate of the allele frequency in the LLFS population.

## Results

The average age of LLFS participants in this study was 71 ± 16 years, and women comprised 55% of all participants. Among 1372 variants identified in the 25 genes, 283 nonsynonymous and stop-gain variants were identified for further review after excluding common variants (variants present in > 0.5% of the general population). Of the 283 variants, seven (2.4%) were stop-gain variants and 276 (97.6%) were nonsynonymous variants. Nine variants (3.2%) were classified as likely pathogenic or pathogenic, 241 variants (85.1%) were classified as variants of uncertain significance (VUS), and the remaining variants were classified as likely benign (11.7%).

All 9 variants classified via ACMG guidelines as likely pathogenic or pathogenic appeared in LLFS at similar frequencies to general population frequencies (Table 1). These variants were present in both autosomal dominant (*BRCA1* and *SDHC*) and autosomal recessive (*CPT2*, *ACADM*, *SUMF1*, *WRN*, *ATM*, and *ACADVL*) genes (Table 1).

**Table 1:** Pathogenic and likely pathogenic classified variants identified in Long Life Family Study

SNP ID^*^	Gene	Amino Acid Change	Random LLFS Sampling Frequency	Population Frequency	***p***-value	ACMG Variant Classification: Criteria^*^	Number of LLFS Participants with Variant
rs28897686: NM_007294.3:c.3748G > T	*BRCA1*	p.Glu1250*	2.57 × 10^−4^	7.90 × 10^−6^	1	P: PVS1, PM2, PP5	3 heterozygotes
rs764575966: NM_003001.3:c.397C > T	*SDHC*	p.Arg133*	7.71 × 10^−4^	3.20 × 10^−5^	0.05	P: PVS1, PM2, PP5	3 heterozygotes
rs74315294: NM_000098.3:c.338C > T	*CPT2*	p.Ser113Leu	3.96 × 10^−3^	1.38 × 10^− 3^	0.11	P: PM1, PM2, PP3, PP5	391 heterozygotes 3 homozygotes
rs121434280: NM_001127328.2:c.211 T > C	*ACADM*	p.Tyr71His	2.06 × 10^−4^	4.87 × 10^− 4^	1	LP: PS3, PM2, PP5, BP4	136 heterozygotes
rs201375579: NM_001127328.2:c.809A > G	*ACADM*	p.Asp270Gly	4.30 × 10^−5^	2.97 × 10^−4^	1	LP: PS3, PM2	79 heterozygotes
rs138058572: NM_001270447.1:c.1427G > A	*ACADVL*	p.Arg476Gln	2.98 × 10^−4^	7.20 × 10^−6^	N/A	LP: PS4, PM2, PP3	1 heterozygote
rs28904921: NM_000051.3:c.7271 T > G	*ATM*	p.Val2424Gly	2.20 × 10^−5^	5.06 × 10^− 5^	1	LP: PS3, PM2,PP3,PP5	14 heterozygotes
rs137852849: NM_001164675.1:c.836C > T	*SUMF1*	p.Ala279Val	2.24 × 10^−4^	1.12 × 10^− 4^	1	LP: PS3, PM2,PP5	31 heterozygotes
rs17847577: NM_000553.4:c.1105C > T	*WRN*	p.Arg369*	1.73 × 10^−4^	1.88 × 10^− 4^	1	P: PVS1, PM2, PP5	53 heterozygotes

^*^P Pathogenic, *LP* Likely Pathogenic

PVS1: nonsense (stop-gain) mutation where loss of function is a known mechanism of disease

PM2: Low allele frequency in genomic databases

PP5: Reputable source(s) classified variant as pathogenic

PS3: Well-established in vitro or in vivo functional studies supportive of a damaging effect on the gene or gene product

PM1: Located in a mutational hot spot and/or critical and well-established functional domain (e.g., active site of an enzyme) without benign variation

PP3: Multiple lines of computational evidence support a deleterious effect on the gene or gene product (conservation, evolutionary, splicing impact, etc.)

BP4: Multiple lines of computational evidence suggest no impact on gene or gene product (conservation, evolutionary, splicing impact, etc.)

PS4: The prevalence of the variant in affected individuals is significantly increased compared with the prevalence in controls

**In-silico algorithms used for PP3 criterion:**

*CPT2* NM_000098.3:c.338C > T: Pathogenic prediction from multiple computational programs (PhyloP: C; SIFT: D; PolyPhen-2: D; Likelihood Ratio Test (LRT): D; MutationTaster: D; GERP: D)

*ACADM* NM_001127328.2:c.211 T > C: Benign prediction from multiple computational programs (PhyloP: C; SIFT: T; PolyPhen-2: B; Likelihood Ratio Test (LRT): N; MutationTaster: D; GERP: D)

A*CADVL* NM_001270447.1:c.1427G > A: Pathogenic prediction from multiple computational programs (PhyloP: C; SIFT: D; PolyPhen-2: D; Likelihood Ratio Test (LRT): D; MutationTaster: D; GERP: D)

*ATM* NM_000051.3:c.7271 T > G: Pathogenic prediction from multiple computational programs (PhyloP: C; SIFT: D; PolyPhen-2: D; Likelihood Ratio Test (LRT): D; MutationTaster: N; GERP: D)

PhyloP: Conserved (C) or not conserved (N); SIFT: Deleterious (D) or tolerated (T); PolyPhen-2: Damaging (D), possibly damaging (P), or benign (B); Likelihood Ratio Test (LRT): Deleterious (D) or neutral (N); MutationTaster: Deleterious (D) or neutral (N); GERP: Deleterious (D) or neutral (N)

Among LLFS participants, the pathogenic *BRCA1* variant (NM_007294.3:c.3748G > T) had an allele frequency similar to the general population (0.03% vs. 0.0008%; *p* = 1) (Table 1). However, the individuals (all heterozygotes), two members of one family (92 year old father and his 49 year old daughter) and one a member of another family (51 year old woman), with this variant present have not been diagnosed with breast, ovarian or prostate cancer. The parental samples of the 92 year old father were not available for evaluation of *BRCA1* mutation status. In the second family, the mother of the 51 year old woman did not carry the *BRCA1* variant. Though the father’s DNA sample was not available for evaluation, we presume this mutation was paternally transmitted though a de novo origin of this mutation cannot be excluded. 18 submissions to ClinVar, ranging from 1994 to 2017 in 657 different individuals from 75 families of varying ethnicities were recorded to have this pathogenic variant in ClinVar in patients with breast or ovarian cancer [27–29]. Though breast and ovarian cancer due to *BRCA1* mutations have a peak incidence in the fourth decade of life, cumulative incidence of these cancers increases till 80 years of age [30].

The prevalence of the *SDHC* variant (NM_003001.3:c.397C > T) in LLFS was marginally higher compared to the general population (0.08% vs. 0.003%; *p* = 0.05) (Table 1). *SDHC* variants are associated with autosomal dominant inherited paraganglioma and gastric stromal sarcoma. The participants from a single family (all heterozygotes) that included a 99 year old mother, her 63 year old daughter and her 55 year old son, were asymptomatic, to the best of our knowledge. Parental samples of the mother were not available for further evaluation. Six submissions to ClinVar, ranging from 2016 to 2017 have listed this particular variant as pathogenic. Among the evidence submitted to ClinVar, this mutation was present in multiple individuals in different families exhibiting paragangliomas [31, 32]. Since hereditary paragangliomas typically present before 45 years of age, the LLFS participants are older than the typical age of onset for hereditary paragangliomas.

The remaining 7 pathogenic/likely pathogenic variants represented variants in genes that caused autosomal recessive disorders and represented carrier status for these autosomal recessive disorders. As expected, these variants were present in frequencies comparable to the general population. These included variants in *CPT2* (*n* = 1), *ACADVL* (*n* = 1), *SUMF1* (*n* = 1), *WRN* (*n* = 1), *ATM* (*n* = 1) and *ACADM* (*n* = 2). Three LLFS participants were homozygous for the pathogenic variant in *CPT2* (rs74315294) (Table 1).

Nonsynonymous or stop-gain mutations variants of uncertain significance were identified in all of the genes studied, except for *FANCI* and are summarized in Supplementary table 2. These variants were unable to be classified as benign or pathogenic because the only evidence available for evaluating biological consequence of these mutations was in-silico predictions from computational programs and prevalence of the variant in the general population, which are not sufficient to classify variants definitively using ACMG criteria.

Variants classified as likely benign (*n* = 33) were found in *WRN* (*n* = 18), *ATM* (*n* = 7), *POLG* (*n* = 4), *GRN* (*n* = 1), *LDLR* (*n* = 2), and *SOD1* (*n* = 1). Thirty-two of these variants were associated with nonsynonymous mutations and one was associated with a stop-gain mutation (Supplementary table 2).

## Discussion

We observed that variants previously classified as pathogenic in both autosomal dominant and autosomal recessive disorders were seen in similar or higher frequencies among individuals from long lived families as compared to the general population. This study sequenced seven (*BRCA1, SDHC, TP53, LMNA, LDLR, PTEN,* and *SMAD3*) of the 59 genes in the ACMG list of incidental findings. This is the first study to systematically evaluate the prevalence of pathogenic mutations in long lived families and tracked transmission of pathogenic mutations across 2 generations within families that demonstrate exceptional longevity. The results from this study is consistent with the finding from the previous study of 44 Ashkenazi Jewish centenarians; in both studies, the frequencies of pathogenic variants are not significantly different from those of the general population [11]. In the Ashkenazi centenarian study, 130 coding variants in genes associated with degenerative, neoplastic and cardiac diseases classified as pathogenic or likely pathogenic were identified [11]. Though participants in this study are younger than participants in centenarian studies, the results from this study are also consistent with previous studies on centenarians that showed that centenarians without phenotypic evidence of specific diseases have similar prevalence of variants associated with complex chronic diseases as the general population [33]. In addition, the results from this study are also consistent with genomic sequencing performed in general risk populations [34, 35] that have shown that pathogenic mutations can be observed in individuals without any evidence of clinical disease. Furthermore, other studies have reported pathogenic mutations in *BRCA1* in women without a family history of breast cancer and pathogenic mutations in *SCN5A* or *KCNH2 in patients without phenotypic and electrophysiological evidence of cardiac arrhythmias* [36, 37]. These findings suggest that established pathogenic variants may have incomplete penetrance in individuals without a strong family history of the specific disorder.

The ACMG list of reportable genes is associated with monogenic diseases that have an intervention available to prevent the disease or lessen its symptoms [7, 8]. However, a recent ACMG guideline on return of secondary genetic findings has clarified that the ACMG list of 59 genes recommended for reporting of incidental genetic findings was intended to be applied only in the context of clinical whole exome/genome sequencing and not intended to be applied for general population screening [38]. Consistent with the ACMG position and results from previous studies, this study show at least two pathogenic variants in genes associated with autosomal dominant conditions, *BRCA1* and *SDHC*, that did not result in overt disease in the LLFS participants. In addition, three LLFS participants were homozygous for the *CPT2* variant, rs74315294, which is the most commonly identified mutation in people with CPT II deficiency that can be characterized by recurrent episodes of myalgia and weakness [39]. These three LLFS participants also did not specifically report muscle weakness, although they were not adequately assessed for this particular phenotype. Despite the ACMG guidelines on return of secondary genetic findings [7, 8], there is no clear consensus among healthcare professionals regarding return of incidental findings of pathogenic variants [40]. Findings from this study and previous studies have significant implications for expanding large scale sequencing efforts to the general population without a strong family history of specific diseases.

This study has several strengths and limitations. Compared to other studies on long lived individuals, the large sample size and the generalizability of the study findings to people of broad European descent are significant strengths of this study, though these findings may not be generalizable to individuals of non-European ancestry. Furthermore, since several LLFS participants with pathogenic mutations were relatively young, the diseases associated with the autosomal dominant diseases might not have manifested yet. For example, the participants with the *BRCA1* variant were in their sixties, which is still relatively young in terms of clinical manifestation of genetic breast or ovarian cancer. A previous study has shown that though the peak incidence of breast cancer was among women in their 40s and 50s, the cumulative risk of breast and ovarian cancer risk increased till 80 years [30]. Thus, these LLFS participants could develop breast or ovarian cancer in the future. Follow-up of LLFS participants over ~ 8 years showed that the 92 year old father with the *BRCA1* mutation and the 99 year old mother with the *SDHC* mutation died of non-cancer related causes while the remaining LLFS participants are alive and have not reported any new diagnosis of cancer during the intervening years. The lack of any family history of autosomal dominant diseases in these families suggests that the pathogenic mutations may not be fully penetrant in these individuals and is these results lend further support to the recent clarification of the ACMG guidelines on reporting secondary genetic findings in the context of general population screening efforts. Despite the large sample size, the low population prevalence of these variants may limit estimation of differences in population prevalence for some variants in LLFS as compared to general population based databases. For example, this study had only 78% power (α = 0.05) to detect differences in population frequency for the *BRCA1* variant (frequency = 0.03%), while this study had 87% power (α = 0.05) to detect differences in population frequency for the *SDHC* variant (frequency = 0.08%). Future studies that extend these results beyond the 25 genes to all 59 genes listed in the ACMG secondary findings list in other large, more diverse long-lived populations and extending our results will further clarify the penetrance of pathogenic mutations in populations without a strong family history of specific diseases.

## Conclusions

The study results suggest that penetrance of pathogenic variants may be lower in a general population as compared to targeted patient populations with a strong family history of specific Mendelian diseases. The lack of overt disease in two generations of family members with pathogenic mutations in this study further supports the idea that populations without a strong family history of specific conditions may demonstrate incomplete penetrance of pathogenic variants. These findings suggest that family history of specific diseases should be an important consideration in deciding whether specific incidental genetic findings should be returned to research participants to minimize potential harm while maximizing patient benefit. Hence, before expanding large scale genomic testing to asymptomatic individuals in the broader community, additional research is needed to better understand genotype-phenotype associations and penetrance of genetic variants in the general population.

## Supplementary information

**Additional file 1: Table S1.** Genes evaluated for previously classified pathogenic mutations.

**Additional file 2: Table S2.** List of variants of uncertain significance found in the Long Life Family Study.

## Data Availability

The datasets generated and/or analyzed during the current study are available in the dbGAP repository, https://www.ncbi.nlm.nih.gov/projects/gap/cgi-bin/study.cgi?study_id=phs000397.v1.p1

## Abbreviations

ACMG: American College of Medical Genetics and Genomics
NGS: Next Generation Sequencing
LLFS: Long Life Family Study
ESP: Exome Sequencing Project
LOVD: Leiden Open Variation Database
GERP: Genomic Evolutionary Rate Profiling
LRT: Likelihood Ratio Test
SIFT: Sorting Intolerant From Tolerant
PolyPhen-2: Polymorphism Phenotyping v2
phyloP100way: Phylogenetic *p* value
gnomAD: Genome Aggregation Database
ANNOVAR: Annotate Variation
