# Supplementary material for: Prevalence of clinically actionable disease variants in exceptionally long-lived families
Source: BMC Med Genomics. 2020 Apr 10;13:61. doi: 10.1186/s12920-020-0710-5 (PMC7146901; doi:10.1186/s12920-020-0710-5)
Supplement: Supplementary file 1 — Additional file 1: Table S1. Genes evaluated for previously classified pathogenic mutations. [file 12920_2020_710_MOESM1_ESM.pdf]

Supplementary Table 1: Genes evaluated for previously classified pathogenic mutations.

Sequences from 25 genes associated with pathogenesis of inborn errors of metabolism, familial cardiovascular disease, familial cancer and neurodegenerative conditions were studied. Rows that are bolded were genes in which likely pathogenic or pathogenic variants were found.

Gene	Syndrome	Inheritance Pattern
BRCA1	**Familial breast cancer**	**Autosomal dominant**
GRN	Frontotemporal lobar degeneration<br>Neuronal ceroid lipofuscinosis	Autosomal dominant<br>Autosomal recessive
SDHC	**Paraganglioma and gastric stromal sarcoma**	**Autosomal dominant**
TERC	Dyskeratosis congenita	Autosomal dominant
TERT	Dyskeratosis congenita	Autosomal dominant/recessive
LDLR	Familial hypercholesterolemia	Autosomal dominant
LMNA	Emery-Dreifuss muscular dystrophy	Autosomal dominant/recessive
LMX1B	Nail Patella syndrome	Autosomal dominant
MAPT	Frontotemporal dementia	Autosomal dominant
PAX2	Glomerulosclerosis, focal segmental, 7	Autosomal dominant
PAX3	Craniofacial-deafness-hand syndrome<br>Waardenburg syndrome	Autosomal dominant<br>Autosomal dominant/recessive
PTEN	Various cancer syndromes	Autosomal dominant
SMAD3	Loeys-Dietz syndrome, type 3	Autosomal dominant
SOD1	Amyotrophic lateral sclerosis 1	Autosomal dominant/recessive
TP53	Li Fraumeni syndrome	Autosomal dominant
ACADM	**Medium chain Acyl-CoA dehydrogenase deficiency**	**Autosomal recessive**
ACADVL	**VLCAD deficiency**	**Autosomal recessive**
ATM	**Ataxia-telangiectasia**	**Autosomal recessive**
BIN1	Centronuclear myopathy	Autosomal recessive
CPT2	**Carnitine palmitoyltransferase deficiency II**	**Autosomal recessive**
FANCI	Fanconi anemia	Autosomal recessive
LARGE	Walker-Warburg syndrome	Autosomal recessive
POLG	Mitochondrial DNA depletion syndrome	Autosomal recessive
SUMF1	**Multiple sulfatase deficiency**	**Autosomal recessive**
WRN	**Werner syndrome**	**Autosomal recessive**
