# Supplementary material for: Prevalence of clinically actionable disease variants in exceptionally long-lived families
Source: BMC Med Genomics. 2020 Apr 10;13:61. doi: 10.1186/s12920-020-0710-5 (PMC7146901; doi:10.1186/s12920-020-0710-5)
Supplement: Supplementary file 2 — Additional file 2: Table S2. List of variants of uncertain significance found in the Long Life Family Study. [file 12920_2020_710_MOESM2_ESM.pdf]

Supplementary Table 2: List of variants of uncertain significance found in the Long Life Family Study .

markname	Gene	# of people in LLFS	Available Evidence for Variant Classification
c1b76198574XXXXXXXXXXXXXXXXXX	*ACADM*	2 heterozygotes	PM2, PP3
c1b76200481XXXXXXXXXXXXXXXXXX	*ACADM*	2 heterozygotes	PM2, PP2, BP4
c1b76205722XXXXXXXXXXXXXXXXXX	*ACADM*	3 heterozygotes	PM2, PP2
c1b76205722XXXXXXXXXXXXXXXXXX	*ACADM*	56 heterozygotes	PM2, PP2
c1b76205728XXXXXXXXXXXXXXXXXX	*ACADM*	4 heterozygotes	PM2, PP2
c1b76205728XXXXXXXXXXXXXXXXXX	*ACADM*	1 heterozygote	PM2, PP2
c1b76211557XXXXXXXXXXXXXXXXXX	*ACADM*	1 homozygote, 35 heterozygotes	PM2, PP2, BP4
c1b76215210XXXXXXXXXXXXXXXXXX	*ACADM*		PM2
c1b76226871XXXXXXXXXXXXXXXXXX	*ACADM*	1 heterozygote	PM2, PP3
c1b76226952XXXXXXXXXXXXXXXXXX	*ACADM*	2 homozygotes, 122 heterozygotes	PM2
c17b7124105XXXXXXXXXXXXXXXXXX	*ACADVL*	7 heterozygotes	PM1, PM2, PP2, BP4
c17b7125564XXXXXXXXXXXXXXXXXX	*ACADVL*		PM2, PP3
c17b7125566XXXXXXXXXXXXXXXXXX	*ACADVL*	5 heterozygotes	PM1, PM2, PP2, BP4
c17b7127674XXXXXXXXXXXXXXXXXX	*ACADVL*	1 homozygote, 119 heterozygotes	PM2, PP2
c17b7127674XXXXXXXXXXXXXXXXXX	*ACADVL*	1 heterozygote	PM2, PP2
c17b7128342XXXXXXXXXXXXXXXXXX	*ACADVL*	99 heterozygotes	PM2, PP2, PP3
rs370146676	*ACADVL*	9 heterozygotes	PM2, PP3
rs200788251	*ACADVL*	33 heterozygotes	PM2, PP3
rs143172658	*ACADVL*	38 heterozygotes	PM2, PP3
c11b108098388XXXXXXXXXXXXXXXX	*ATM*	5 heterozygotes	PM2
c11b108098551XXXXXXXXXXXXXXXX	*ATM*		PM2, BP4
c11b108098563XXXXXXXXXXXXXXXX	*ATM*	2 heterozygotes	PM2, PP3
c11b108098563XXXXXXXXXXXXXXXX	*ATM*	6 heterozygotes	PM2, PP3
c11b108099982XXXXXXXXXXXXXXXX	*ATM*		PM2, BP4
c11b108100014XXXXXXXXXXXXXXXX	*ATM*	71 heterozygotes	PM2
c11b108106433XXXXXXXXXXXXXXXX	*ATM*		PM2
c11b108106463XXXXXXXXXXXXXXXX	*ATM*	1 heterozygote	PM2
c11b108106463XXXXXXXXXXXXXXXX	*ATM*	1 heterozygote	PM2
c11b108115601XXXXXXXXXXXXXXXX	*ATM*	1 homozygote, 12 heterozygotes	PM2, BP4
c11b108117837XXXXXXXXXXXXXXXX	*ATM*	2 heterozygotes	PM2, PP3
c11b108117837XXXXXXXXXXXXXXXX	*ATM*	1 heterozygote	PM2, PP3
c11b108121787XXXXXXXXXXXXXXXX	*ATM*	67 heterozygotes	PM2, PP3
c11b108121787XXXXXXXXXXXXXXXX	*ATM*	1 heterozygote	PM2, PP3
c11b108122645XXXXXXXXXXXXXXXX	*ATM*	1 heterozygote	PM2, BP4
c11b108122659XXXXXXXXXXXXXXXX	*ATM*	9 heterozygotes	PM2, BP4
c11b108122659XXXXXXXXXXXXXXXX	*ATM*	53 heterozygotes	PM2, BP4
c11b108124663XXXXXXXXXXXXXXXX	*ATM*	45 heterozygotes	PM2, BP4
c11b108126966XXXXXXXXXXXXXXXX	*ATM*	12 heterozygotes	PM2
c11b108126967XXXXXXXXXXXXXXXX	*ATM*	5 heterozygotes	PM2
c11b108126967XXXXXXXXXXXXXXXX	*ATM*	3 heterozygotes	PM2
c11b108137923XXXXXXXXXXXXXXXX	*ATM*	1 heterozygote	PM2, BP4
c11b108137925XXXXXXXXXXXXXXXX	*ATM*	3 heterozygotes	PM2, BP4
c11b108137925XXXXXXXXXXXXXXXX	*ATM*	68 heterozygotes	PM2, BP4
c11b108139269XXXXXXXXXXXXXXXX	*ATM*	19 heterozygotes	PM2, PP3
c11b108153536XXXXXXXXXXXXXXXX	*ATM*	3 heterozygotes	PM2
c11b108158393XXXXXXXXXXXXXXXX	*ATM*	54 heterozygotes	PM2, BP4
c11b108158399XXXXXXXXXXXXXXXX	*ATM*	39 heterozygotes	PM2, BP4
c11b108160416XXXXXXXXXXXXXXXX	*ATM*	84 heterozygotes	PM2, PP3, BP6
c11b108160467XXXXXXXXXXXXXXXX	*ATM*	12 heterozygotes	PM2, PP3
c11b108160516XXXXXXXXXXXXXXXX	*ATM*	158 heterozygotes	PM2, BP6

c11b108163354XXXXXXXXXXXXXXXX	*ATM*	4 heterozygotes	PM2, BP4
c11b108172425XXXXXXXXXXXXXXXX	*ATM*	5 heterozygotes	PM2, PP5
c11b108172459XXXXXXXXXXXXXXXX	*ATM*	5 heterozygotes	PM2, PP3
c11b108172475XXXXXXXXXXXXXXXX	*ATM*	5 heterozygotes	PM2
c11b108173678XXXXXXXXXXXXXXXX	*ATM*	4 heterozygotes	PM2, BP4
c11b108173701XXXXXXXXXXXXXXXX	*ATM*	1 heterozygote	PM2, PP3
c11b108178632XXXXXXXXXXXXXXXX	*ATM*	8 heterozygotes	PM2, BP4
c11b108178699XXXXXXXXXXXXXXXX	*ATM*	3 heterozygotes	PM2, PP3
c11b108180889XXXXXXXXXXXXXXXX	*ATM*	1 heterozygote	PM2
c11b108180898XXXXXXXXXXXXXXXX	*ATM*	1 heterozygote	PM2
c11b108180916XXXXXXXXXXXXXXXX	*ATM*		PM2, BP4
c11b108180945XXXXXXXXXXXXXXXX	*ATM*	30 heterozygotes	PM2
c11b108181014XXXXXXXXXXXXXXXX	*ATM*	1 homozygote, 24 heterozygotes	PM2, BP4
c11b108183194XXXXXXXXXXXXXXXX	*ATM*	1 homozygote, 93 heterozygotes	PM2, BP4
c11b108183194XXXXXXXXXXXXXXXX	*ATM*	2 heterozygotes	PM2, BP4
c11b108186743XXXXXXXXXXXXXXXX	*ATM*	37 heterozygotes	PM2, PP3
c11b108188216XXXXXXXXXXXXXXXX	*ATM*	24 heterozygotes	PM2, PP3
c11b108196908XXXXXXXXXXXXXXXX	*ATM*		PM2
c11b108199947XXXXXXXXXXXXXXXX	*ATM*		PM2
c11b108200997XXXXXXXXXXXXXXXX	*ATM*		PM2, PP3
c11b108201023XXXXXXXXXXXXXXXX	*ATM*	118 heterozygotes	PM2
c11b108201108XXXXXXXXXXXXXXXX	*ATM*	27 heterozygotes	PM2, PP3
c11b108202754XXXXXXXXXXXXXXXX	*ATM*	21 heterozygotes	PM2
c11b108203516XXXXXXXXXXXXXXXX	*ATM*		PM2, BP4
c11b108206680XXXXXXXXXXXXXXXX	*ATM*		PM2, PP3
c11b108213968XXXXXXXXXXXXXXXX	*ATM*	1 heterozygote	PM2
c11b108216546XXXXXXXXXXXXXXXX	*ATM*	31 heterozygotes	PM2
c11b108224508XXXXXXXXXXXXXXXX	*ATM*		PM2, PP3
c11b108235926XXXXXXXXXXXXXXXX	*ATM*	13 heterozygotes	PM2
c11b108236150XXXXXXXXXXXXXXXX	*ATM*	40 heterozygotes	PM2
c11b108236150XXXXXXXXXXXXXXXX	*ATM*	1 heterozygote	PM2
c2b127806143XXXXXXXXXXXXXXXXX	*BIN1*	1 homozygote, 14 heterozygotes	PM2, PP3
c2b127821511XXXXXXXXXXXXXXXXX	*BIN1*	115 heterozygotes	PM2, PP3, BP6
c1b53675846XXXXXXXXXXXXXXXXXX	*CPT2*	56 heterozygotes	PM1, PM2
c1b53675960XXXXXXXXXXXXXXXXXX	*CPT2*	9 heterozygotes	PP3
c1b53676011XXXXXXXXXXXXXXXXXX	*CPT2*	1 heterozygote	PM1, PM2
c1b53676067XXXXXXXXXXXXXXXXXX	*CPT2*	36 heterozygotes	PM1, PM2
c1b53676085XXXXXXXXXXXXXXXXXX	*CPT2*	1 heterozygote	PM1, PM2
c1b53676146XXXXXXXXXXXXXXXXXX	*CPT2*	3 heterozygotes	PM1, PM2
c1b53676172XXXXXXXXXXXXXXXXXX	*CPT2*	15 heterozygotes	PM1, PM2
c1b53676199XXXXXXXXXXXXXXXXXX	*CPT2*	12 heterozygotes	PM1, PM2
c1b53676267XXXXXXXXXXXXXXXXXX	*CPT2*	12 heterozygotes	PM2, BP4
c1b53676371XXXXXXXXXXXXXXXXXX	*CPT2*	327 heterozygotes	PP3
c1b53676548XXXXXXXXXXXXXXXXXX	*CPT2*		PM1, PM2, PP2
c1b53676688XXXXXXXXXXXXXXXXXX	*CPT2*	52 heterozygotes	PM2, PP3, BP2
c1b53676782XXXXXXXXXXXXXXXXXX	*CPT2*	4 heterozygotes	PM2, PP2
c1b53676782XXXXXXXXXXXXXXXXXX	*CPT2*	13 heterozygotes	PM2, PP2
c1b53676869XXXXXXXXXXXXXXXXXX	*CPT2*		PM2
c1b53679140XXXXXXXXXXXXXXXXXX	*CPT2*	1 heterozygote	PM1, PM2, PP2
c15b89859652XXXXXXXXXXXXXXXXX	*FANCI*	1 heterozygote	PM2
c17b42426594XXXXXXXXXXXXXXXXX	*GRN*	1 heterozygote	PM2, PP3
c17b42426834XXXXXXXXXXXXXXXXX	*GRN*	2 heterozygotes	PM2,BP4
c17b42426863XXXXXXXXXXXXXXXXX	*GRN*	15 heterozygotes	PM2, PP3
c17b42426863XXXXXXXXXXXXXXXXX	*GRN*	1 heterozygote	PM2, PP3

c17b42426906XXXXXXXXXXXXXXXXX	*GRN*	1 heterozygote	PM2, PP3
c17b42427099XXXXXXXXXXXXXXXXX	*GRN*	22 heterozygotes	PM2, BP4
c17b42427605XXXXXXXXXXXXXXXXX	*GRN*	4 homozygotes, 278 heterozygotes	PM2, BP4
c17b42428080XXXXXXXXXXXXXXXXX	*GRN*	2 heterozygotes	PM2, BP4
c17b42428080XXXXXXXXXXXXXXXXX	*GRN*	6 heterozygotes	PM2, BP4
c17b42428080XXXXXXXXXXXXXXXXX	*GRN*	1 heterozygote	PM2, BP4
c17b42428499XXXXXXXXXXXXXXXXX	*GRN*	5 heterozygotes	PM2
c17b42429003XXXXXXXXXXXXXXXXX	*GRN*	24 heterozygotes	PM2, BP4
c17b42429396XXXXXXXXXXXXXXXXX	*GRN*	15 heterozygotes	PM2
c17b42429835XXXXXXXXXXXXXXXXX	*GRN*	13 heterozygotes	PM2, BP4
c17b42429898XXXXXXXXXXXXXXXXX	*GRN*	1 heterozygote	PM2
c17b42429898XXXXXXXXXXXXXXXXX	*GRN*	3 heterozygotes	PM2
c17b42430047XXXXXXXXXXXXXXXXX	*GRN*	1 heterozygote	PM2
c17b42430047XXXXXXXXXXXXXXXXX	*GRN*	14 heterozygotes	PM2
c17b42430097XXXXXXXXXXXXXXXXX	*GRN*		PM2
c22b33670513XXXXXXXXXXXXXXXXX	*LARGE*	1 heterozygote	PM2, PP3
c22b33670513XXXXXXXXXXXXXXXXX	*LARGE*	8 heterozygotes	PM2, PP3
c22b33673227XXXXXXXXXXXXXXXXX	*LARGE*	33 heterozygotes	PM2
c22b33679263XXXXXXXXXXXXXXXXX	*LARGE*	2 heterozygotes	PM2
c22b33700300XXXXXXXXXXXXXXXXX	*LARGE*	9 heterozygotes	PM2, PP3
c22b33780242XXXXXXXXXXXXXXXXX	*LARGE*	11 heterozygotes	PM2, PP3
c22b33828243XXXXXXXXXXXXXXXXX	*LARGE*	19 heterozygotes	PM2
c22b34022295XXXXXXXXXXXXXXXXX	*LARGE*	4 heterozygotes	PM2, BP4
c22b34046370XXXXXXXXXXXXXXXXX	*LARGE*	1 homozygote, 136 heterozygotes	PM2, BP4
c22b34046501XXXXXXXXXXXXXXXXX	*LARGE*	2 heterozygotes	PM2, BP4
c22b34046501XXXXXXXXXXXXXXXXX	*LARGE*	3 heterozygotes	PM2, BP4
c22b34046583XXXXXXXXXXXXXXXXX	*LARGE*	46 heterozygotes	PM2
c19b11213381XXXXXXXXXXXXXXXXX	*LDLR*	8 heterozygotes	PM2, PP3, PP5
c19b11221390XXXXXXXXXXXXXXXXX	*LDLR*	8 heterozygotes	PM2, PP3
c19b11221390XXXXXXXXXXXXXXXXX	*LDLR*	1 heterozygote	PM2, PP3
c19b11221414XXXXXXXXXXXXXXXXX	*LDLR*	7 heterozygotes	PM2, PP3
c19b11224435XXXXXXXXXXXXXXXXX	*LDLR*	1 heterozygote	PM2
c19b11227631XXXXXXXXXXXXXXXXX	*LDLR*		PM2, PP3
c19b11230798XXXXXXXXXXXXXXXXX	*LDLR*	85 heterozygotes	PM2, BP6
c19b11231049XXXXXXXXXXXXXXXXX	*LDLR*		PM2, BP4
c19b11231084XXXXXXXXXXXXXXXXX	*LDLR*	3 heterozygotes	PM2, PP3
c19b11231084XXXXXXXXXXXXXXXXX	*LDLR*	1 heterozygote	PM2, PP3
c19b11231156XXXXXXXXXXXXXXXXX	*LDLR*	9 heterozygotes	PM2, PP5
c19b11231164XXXXXXXXXXXXXXXXX	*LDLR*	36 heterozygotes	PM2, BP6
c19b11231164XXXXXXXXXXXXXXXXX	*LDLR*	1 heterozygote	PM2
c19b11231174XXXXXXXXXXXXXXXXX	*LDLR*	1 heterozygote	PM2, BP4
c19b11233915XXXXXXXXXXXXXXXXX	*LDLR*	5 heterozygotes	PM2, BP4
c19b11233915XXXXXXXXXXXXXXXXX	*LDLR*	1 heterozygote	PM2, BP4
c19b11233915XXXXXXXXXXXXXXXXX	*LDLR*	1 heterozygote	PM2, BP4
c19b11233940XXXXXXXXXXXXXXXXX	*LDLR*	1 homozygote, 226 heterozygotes	PM2, BP4
c19b11233961XXXXXXXXXXXXXXXXX	*LDLR*	23 heterozygotes	PM2, BP4, BP6
c19b11234016XXXXXXXXXXXXXXXXX	*LDLR*	9 heterozygotes	PM2, BP4
c19b11240278XXXXXXXXXXXXXXXXX	*LDLR*	1 homozygote, 252 heterozygotes	PM2, PP3, BP6
c1b156105728XXXXXXXXXXXXXXXXX	*LMNA*		PM2, PP3
c1b156107470XXXXXXXXXXXXXXXXX	*LMNA*	43 heterozygotes	PM2, PP2, PP3
c1b156108880XXXXXXXXXXXXXXXXX	*LMNA*	35 heterozygotes	PM2, PP3
c9b129458653XXXXXXXXXXXXXXXXX	*LMX1B*	6 heterozygotes	PM2, PP2
c9b129458692XXXXXXXXXXXXXXXXX	*LMX1B*	11 heterozygotes	PM2, PP2, PP3
c17b44039765XXXXXXXXXXXXXXXXX	*MAPT*	9 heterozygotes	PM2

c17b44039824XXXXXXXXXXXXXXXXX	*MAPT*	10 heterozygotes	PM2, BP4
c17b44055753XXXXXXXXXXXXXXXXX	*MAPT*	13 heterozygotes	PM2, PP3
c17b44055753XXXXXXXXXXXXXXXXX	*MAPT*	3 heterozygotes	PM2, PP3
c17b44061015XXXXXXXXXXXXXXXXX	*MAPT*	47 heterozygotes	PM2
c17b44061185XXXXXXXXXXXXXXXXX	*MAPT*	1 heterozygote	PM2, BP4
c17b44064418XXXXXXXXXXXXXXXXX	*MAPT*	61 heterozygotes	PM2, BP4
c17b44067289XXXXXXXXXXXXXXXXX	*MAPT*	24 heterozygotes	PM2
c10b102566298XXXXXXXXXXXXXXXX	*PAX2*	148 heterozygotes	PM1, PM2, PP3, BP1
c10b102566298XXXXXXXXXXXXXXXX	*PAX2*	4 heterozygotes	PM1, PM2, PP3, BP1
c10b102566298XXXXXXXXXXXXXXXX	*PAX2*	2 heterozygotes	PM1, PM2, PP3, BP1
c10b102568941XXXXXXXXXXXXXXXX	*PAX2*	72 heterozygotes	PM1, PM2, BP1
c10b102568941XXXXXXXXXXXXXXXX	*PAX2*	1 homozygote, 6 heterozygotes	PM1, PM2, BP1
c10b102586818XXXXXXXXXXXXXXXX	*PAX2*	1 heterozygote	PM2, BP1
c10b102587372XXXXXXXXXXXXXXXX	*PAX2*	11 heterozygotes	PM1, PM2, PP3, BP1
c2b223066148XXXXXXXXXXXXXXXXX	*PAX3*	2 heterozygotes	PM2
c2b223066148XXXXXXXXXXXXXXXXX	*PAX3*	2 heterozygotes	PM2
c2b223066656XXXXXXXXXXXXXXXXX	*PAX3*		PM2
c2b223084894XXXXXXXXXXXXXXXXX	*PAX3*	1 heterozygote	PM1, PM2
c2b223084914XXXXXXXXXXXXXXXXX	*PAX3*	62 heterozygotes	PM1, PM2, PP3
c2b223086010XXXXXXXXXXXXXXXXX	*PAX3*		PM2, BP4
c2b223096868XXXXXXXXXXXXXXXXX	*PAX3*	1 homozygote, 106 heterozygotes	PM1, PM2, PP3
c2b223160316XXXXXXXXXXXXXXXXX	*PAX3*	14 heterozygotes	PM2
c15b89860002XXXXXXXXXXXXXXXXX	*POLG*	1 heterozygote	PM2, BP4
c15b89860745XXXXXXXXXXXXXXXXX	*POLG*	2 heterozygotes	PM2, PP3
c15b89861811XXXXXXXXXXXXXXXXX	*POLG*	2 heterozygotes	PM2
c15b89861833XXXXXXXXXXXXXXXXX	*POLG*	8 heterozygotes	PM2, PP3
c15b89861872XXXXXXXXXXXXXXXXX	*POLG*	7 heterozygotes	PM2, PP3, PP5
c15b89862193XXXXXXXXXXXXXXXXX	*POLG*	2 heterozygotes	PM2, PP3
c15b89862193XXXXXXXXXXXXXXXXX	*POLG*	3 heterozygotes	PM2, PP3
c15b89862268XXXXXXXXXXXXXXXXX	*POLG*		PM2, PP3
c15b89862295XXXXXXXXXXXXXXXXX	*POLG*	2 heterozygotes	PM2, PP3
c15b89862465XXXXXXXXXXXXXXXXX	*POLG*	66 heterozygotes	PM2, BP4
c15b89862465XXXXXXXXXXXXXXXXX	*POLG*	1 heterozygote	PM2, BP4
c15b89862549XXXXXXXXXXXXXXXXX	*POLG*		PM2, PP3
c15b89862577XXXXXXXXXXXXXXXXX	*POLG*	8 heterozygotes	PM2, PP3
c15b89864992XXXXXXXXXXXXXXXXX	*POLG*	6 heterozygotes	PM2, PP3
c15b89866654XXXXXXXXXXXXXXXXX	*POLG*	20 heterozygotes	PM2, PP3
c15b89866693XXXXXXXXXXXXXXXXX	*POLG*	1 homozygote, 96 heterozygotes	PM2, PP3
c15b89867424XXXXXXXXXXXXXXXXX	*POLG*	116 homozygotes, 2418 heterozygotes	PM2, BP4
c15b89869956XXXXXXXXXXXXXXXXX	*POLG*	3 heterozygotes	PM2
c15b89870235XXXXXXXXXXXXXXXXX	*POLG*	1 heterozygote	PM2, PP3
c15b89870235XXXXXXXXXXXXXXXXX	*POLG*	12 heterozygotes	PM2, PP3
c15b89870429XXXXXXXXXXXXXXXXX	*POLG*	134 heterozygotes	PM2, BP4
c15b89870489XXXXXXXXXXXXXXXXX	*POLG*	1 heterozygote	PM2, PP3
c15b89872227XXXXXXXXXXXXXXXXX	*POLG*	83 heterozygotes	PM2, BP4, BP6
c15b89872227XXXXXXXXXXXXXXXXX	*POLG*	22 heterozygotes	PM2, BP4
c15b89876499XXXXXXXXXXXXXXXXX	*POLG*	6 heterozygotes	PM2, PP3
c15b89867339XXXXXXXXXXXXXXXXX	*POLG;POLG*	13 heterozygotes	PM2
c21b33039659XXXXXXXXXXXXXXXXX	*SOD1*	8 heterozygotes	PM2, BP4
c3b4403848XXXXXXXXXXXXXXXXXXX	*SUMF1*	7 heterozygotes	PM2, BP4
c3b4418030XXXXXXXXXXXXXXXXXXX	*SUMF1*		PM2, PP3
c3b4458816XXXXXXXXXXXXXXXXXXX	*SUMF1*	1 heterozygote	PM2, PM5
c3b4458850XXXXXXXXXXXXXXXXXXX	*SUMF1*	19 heterozygotes	PM2, PP2, BP4

c3b4459766XXXXXXXXXXXXXXXXXXXX	*SUMF1*	3 heterozygotes	PM2, PP3, PP5
c3b4491006XXXXXXXXXXXXXXXXXXXX	*SUMF1*	18 heterozygotes	PM2, PP3, PP5
c3b4491007XXXXXXXXXXXXXXXXXXXX	*SUMF1*		PM2
c3b4494663XXXXXXXXXXXXXXXXXXXX	*SUMF1*	1 heterozygote	PM2, BP4
c3b4494683XXXXXXXXXXXXXXXXXXXX	*SUMF1*	9 heterozygotes	PM2, BP4
c3b4494703XXXXXXXXXXXXXXXXXXXX	*SUMF1*	5 heterozygotes	PM2, PP3
c3b4494720XXXXXXXXXXXXXXXXXXXX	*SUMF1*	3 heterozygotes	PM2
c5b1255409XXXXXXXXXXXXXXXXXXXX	*TERT*	23 heterozygotes	PM2, BP4,
c5b1260607XXXXXXXXXXXXXXXXXXXX	*TERT*	1 heterozygote	PM1, PM2, BP4
c8b30916678XXXXXXXXXXXXXXXXXXX	*WRN*	9 heterozygotes	PM2, BP1
c8b30916702XXXXXXXXXXXXXXXXXXX	*WRN*	94 heterozygotes	PM2, BP1
c8b30921824XXXXXXXXXXXXXXXXXXX	*WRN*	1 heterozygote	PM2, BP1
c8b30921824XXXXXXXXXXXXXXXXXXX	*WRN*	64 heterozygotes	PM2, BP1
c8b30921944XXXXXXXXXXXXXXXXXXX	*WRN*	14 heterozygotes	PM2, PP3, BP1
c8b30948355XXXXXXXXXXXXXXXXXXX	*WRN*		PM2, PP3, BP1
c8b30948382XXXXXXXXXXXXXXXXXXX	*WRN*	6 heterozygotes	PM2, BP1
c8b30954294XXXXXXXXXXXXXXXXXXX	*WRN*	4 heterozygotes	PM2, PP3, BP1
c8b30954294XXXXXXXXXXXXXXXXXXX	*WRN*	107 heterozygotes	PM2, PP3, BP1
c8b30958412XXXXXXXXXXXXXXXXXXX	*WRN*	1 homozygote, 27 heterozygotes	PM2, PP3, BP1
c8b30969156XXXXXXXXXXXXXXXXXXX	*WRN*	93 heterozygotes	PM2, PP3, BP1
c8b30969257XXXXXXXXXXXXXXXXXXX	*WRN*	7 heterozygotes	PM2, BP1
c8b30969276XXXXXXXXXXXXXXXXXXX	*WRN*	7 heterozygotes	PM2, BP1
c8b30973896XXXXXXXXXXXXXXXXXXX	*WRN*	15 heterozygotes	PM2, PP3, BP1
c8b30973896XXXXXXXXXXXXXXXXXXX	*WRN*	1 heterozygote	PM2, PP3, BP1
c8b30982112XXXXXXXXXXXXXXXXXXX	*WRN*	1 heterozygote	PM2, PP3, BP1
c8b30998964XXXXXXXXXXXXXXXXXXX	*WRN*	113 heterozygotes	PM2, BP1
c8b30998973XXXXXXXXXXXXXXXXXXX	*WRN*	6 heterozygotes	PM2, BP1
c8b30999054XXXXXXXXXXXXXXXXXXX	*WRN*		PM2, PP3, BP1
c8b31004979XXXXXXXXXXXXXXXXXXX	*WRN*	2 heterozygotes	PM2, PP3, BP1
c8b31014968XXXXXXXXXXXXXXXXXXX	*WRN*		PM2, BP4
